# Visual outcomes and patient satisfaction after implantations of three types of presbyopia-correcting intraocular lenses that have undergone corneal refractive surgery

**DOI:** 10.1038/s41598-024-58653-z

**Published:** 2024-04-10

**Authors:** Shuang Ni, Baoxian Zhuo, Lei Cai, Min Wang, Jiying Shen, Limei Zhang, Wenqian Shen, Haike Guo, Jin Yang

**Affiliations:** 1 Department of Ophthalmology, Shanghai Heping Eye Hospital, Shanghai, China; 2 Department of Ophthalmology and the Eye Institute, Eye and Ear, Nose, and Throat Hospital, Fudan University, 83 Fenyang Road, Xuhui Direct, Shanghai, China; 3 The Key Laboratory of Myopia, Ministry of Health, Shanghai, China; 4 Shanghai Key Laboratory of Visual Impairment and Restoration, Shanghai, China

**Keywords:** Lens diseases, Refractive errors

## Abstract

This prospective, non-randomized, comparative study aimed to compare the visual outcomes and patient satisfaction after implantations of three presbyopia-correcting intraocular lenses (IOLs) after myopic refractive surgery. It was conducted from January 2020 to December 2021 in Shanghai Heping Eye Hospital. Patients were divided into three groups based on the type of IOL implanted. The visual acuity, refractive stability, high-order aberrations, objective visual quality, spectacle independence, and visual function index 14 questionnaire scores of the three groups were compared. This study included 78 eyes of 39 patients: 26 eyes with 839MP, 26 eyes with MF30, and 26 eyes with ZXR00. Uncorrected distance visual acuity improved significantly for all three groups. For a pupil diameter of 4.0 mm, the spherical aberrations of the three groups were 0.33 ± 0.16 *μ*, 0.50 ± 0.08 *μ*, and 0.39 ± 0.10 *μ*, respectively. The spectacle independence for distance vision was over 90% in each group; for near vision, it was only 25% for the ZXR00 group. All three types of presbyopia-correcting IOLs improved visual quality in post-LASIK or PRK patients. However, the high incidence of photic phenomena after presbyopia-correcting IOL implantation in patients who have undergone myopic LASIK should not be neglected.

## Introduction

Since the early 1990s, corneal refractive laser surgery has been widely used for ametropia correction globally^1,2^. Patients who choose to undergo myopic laser-assisted in situ keratomileusis (LASIK) or photorefractive keratectomy (PRK) have high expectations for spectacle independence. Consequently, they have a stronger desire than any other population to achieve full vision after surgery when presbyopia or cataract occurs after LASIK or PRK^3^.

Despite the advances in the current surgical techniques and intraocular lens (IOL) designs, most surgeons recommend monofocal IOLs for these patients because of the increase in high-order aberrations (HOAs) induced by LASIK or PRK^4^. Recently, presbyopia-correcting IOLs, such as AT LISA tri 839MP (839MP), Lentis MplusX LS-313 MF30 (MF30), and TECNIS Symfony ZXR00 (ZXR00) have been reported to provide better visual quality and acuity than earlier models^5–7^. Qiu-Mei Li et al. reported the restoration of visual acuity after presbyopic lens extraction with trifocal intraocular lens implantation in eyes with previous myopic or hyperopic corneal laser vision correction^8^. Páez et al. reported a case series of MF30 IOL implantations in 22 patients with myopia who underwent LASIK or PRK with satisfactory outcomes for both distance and near vision^9^. For ZXR00 IOLs, Ferreira et al. found that it was helpful in restoring visual function after cataract surgery in eyes that had previously undergone myopic LASIK surgery, providing visual quality comparable to those achieved with monofocal IOL^10^.

Several reports have compared the differences in visual quality and photic phenomena after implantation of different types of presbyopia-correcting IOLs in patients who have undergone myopic LASIK or PRK^11,12^. However, the most suitable type of IOL for these patients has yet to be ascertained. This study aimed to compare the visual outcomes, patient satisfaction, and photic phenomena after the implantation of three different presbyopia-correcting IOLs (839MP, MF30, and ZXR00) in cataract patients who had undergone corneal refractive surgery. The study will provide clinicians with reference data for the use of presbyopia-correcting IOLs for this population.

## Methods

### Patients

This was a prospective, non-randomized, comparative study conducted at Shanghai Heping Eye Hospital, Shanghai, China, from January 2020 to December 2021. Patients who had undergone myopic LASIK or PRK, had been scheduled for cataract surgery, and desired spectacle independence were enrolled. They were divided into three groups based on the three types of lenses implanted (839MP, MF30, and ZXR00), which were decided by patients themselves. The 839MP, ZXR00, and MF30 IOLs were binocularly implanted in patients who required full visual acuity (VA), intermediate-to-distance VA, and near-to-distance VA, respectively.

Patients diagnosed with cataracts who were aged between 40 and 65 years and had a history of myopic LASIK or PRK within the previous 25 years were included in the study. The exclusion criteria included previous monocular best-corrected distance visual acuity (CDVA) of < 0.1 logarithm of the minimum angle of resolution (LogMAR) before corneal refractive surgery, preoperative corneal astigmatism of more than 1.0 diopter (D), previous intraocular surgery, and ocular pathologies such as macular disease, retinopathy, neuro-ophthalmic disease, corneal disease, and glaucoma. All patients were informed of the study, and they provided informed consent to undergo clinical examinations following the principles of the Declaration of Helsinki. This study was approved by the ethics committee of Shanghai Heping Eye Hospital (HXYK-SHHP-2019-0005).

### Examinations and surgical technique

All patients underwent a complete ophthalmologic examination, including measurement of uncorrected distance visual acuity (UDVA) and best-corrected distance visual acuity (CDVA), manifest refraction, slit-lamp biomicroscopy, tonometry, optical coherence tomography, ray tracing aberrometry (iTrace, Tracey technologies, Texas, USA), optical biometry (IOL-Master 700, Carl Zeiss Meditec AG, Jena, Germany), and corneal topography (Pentacam, Oculus Optikgeraete GmbH, Wetzlar, Germany). Tracy iTrace was used to confirm the regularity of ablation and astigmatism and measure corneal HOAs before implantation. The aberrometer iTrace was utilized for wavefront analysis, employing the ray-tracing principle. It sequentially projects 256 near-infrared laser beams into the eye in a specific scanning pattern, with parameter detection taking less than 200 ms. Topographies were acquired using the Placido-based corneal topographer integrated into the same device. Corneal aberrations were computed using anterior topography data, while internal aberrations were determined by subtracting the corneal wavefront aberrations from those of the entire eye, as measured by the ray-tracing aberrometer using the built-in program.

All cataract surgeries were performed by two experienced surgeons (YANG J, GUO HK) using a 2.2 mm clear corneal incision under topical anesthesia. Phacoemulsification was performed using the Centrion® Vision system (Alcon, Texas, USA), followed by irrigation, aspiration of the cortex, and IOL implantation in the capsular bag using an injector. Surgeries in the other eye followed 7 days later. Postoperative anti-inflammatory and antibiotic agents were administered for 4 weeks.

The data obtained by the IOL master 700 were entered into the Barrett True K formula to calculate the IOL power. The targeted refraction was − 0.2 to − 0.5 D for the 839MP and MF30 groups and − 0.5 to − 0.75 D for the ZXR00 group.

All patients were followed-up at 1 day, 1 week, 1 month, and 3 months after surgery. The visual and refractive outcomes at 3 months were used as the primary endpoints. The postoperative CDVA, UDVA, uncorrected intermediate visual acuity (UIVA), uncorrected near visual acuity (UNVA), subjective manifest absolute error spherical equivalent (AESE), and variations were measured 1 day, 1 week, 1 month, and 3 months after surgery. The modulation transfer function (MTF) curve of the total eye and HOA with 3.0- and 4.0-mm pupils were collected at 3 months postoperatively. Functional vision was assessed using the modified Vision Acuity and Visual Function Index 14 (VF-14) questionnaire at 3 months postoperatively^13^. The patients also completed a questionnaire including frequency, severity, disturbance of the photic phenomena (including halos, starburst, and glare) at night and under mesopic conditions, as well as satisfaction with distance and near vision.

### Statistical analysis

The data were analyzed using SPSS for Windows (version 26.0, IBM Inc., Chicago, IL, USA). The descriptive demographic data, visual and refractive outcomes, visual quality, and spectacle independence were analyzed. The normality of the data distribution was determined using normal probability plots and Kolmogorov–Smirnov tests. When parametric analysis was possible for normally distributed data, one-way analysis of variance (ANOVA) was used with the Bonferroni test for multiple comparisons. Otherwise, the Kruskal–Wallis non-parametric test was performed. Differences were considered statistically significant for P-values less than 0.05.

## Results

### Baseline demographic data

**Figure 1:**
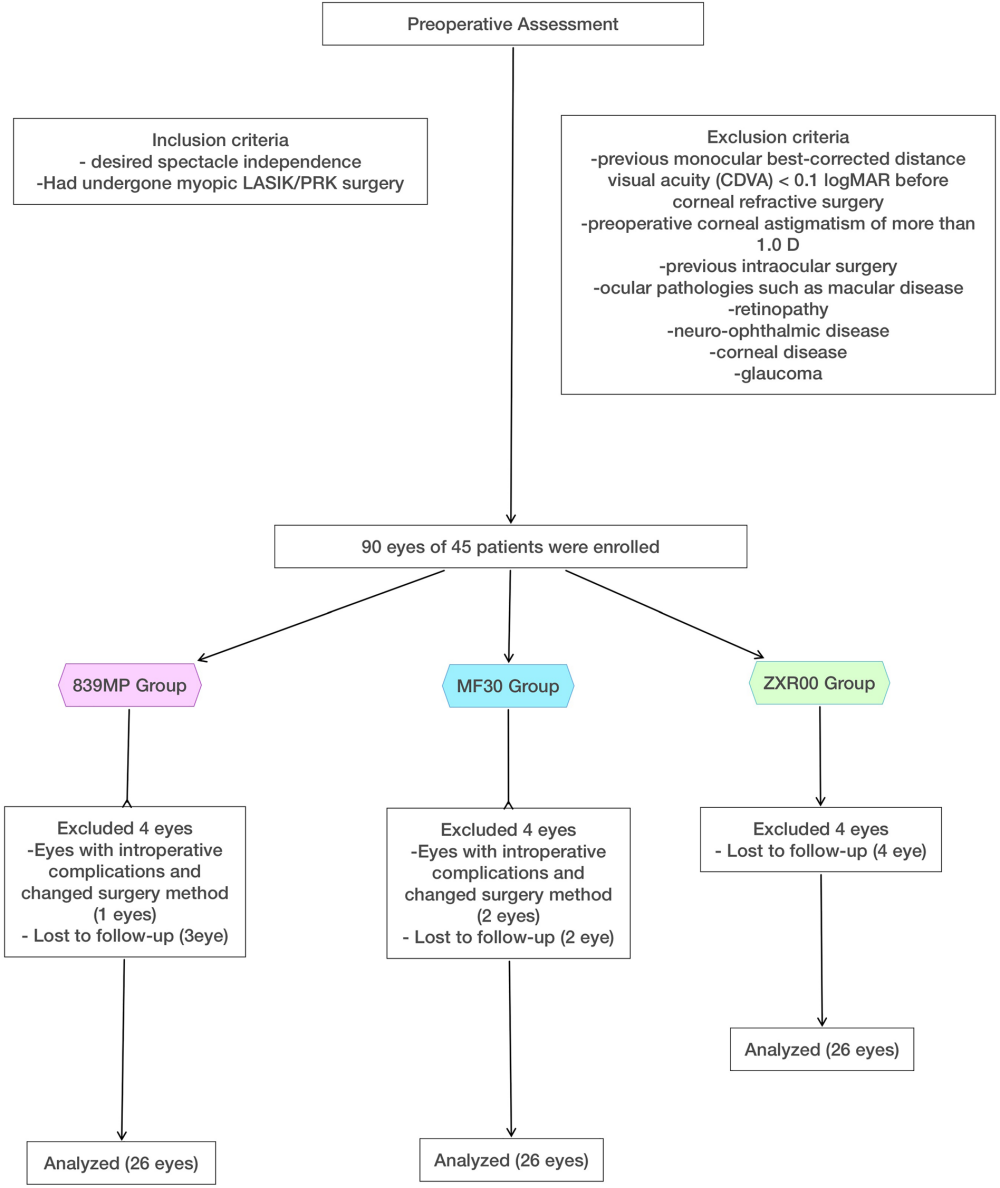
Flowchart of patient enrolment and follow-up.

The study included 90 eyes of 45 patients. Three eyes for which the surgical approach was changed because of intraoperative complications were excluded (1 in the 839MP group and 2 in the MF30 group). Patients with 1 eye available was not included to avoid potential bias. Eight eyes (3 in the 839MP group, 2 in the MF30 group, and 4 in the ZXR00 group) were lost to follow-up. Finally, 78 eyes (26, 26, and 26 eyes in the 839MP, MF30, and ZXR00 groups, respectively) were evaluated (Fig. 1). As shown in Table 1, there were no significant differences in age, axial length (AL), anterior chamber depth, flat keratometry, steep keratometry, preoperative best-corrected visual acuity, and preoperative high-order corneal aberrations (PREHOA cornea) among the three groups (*P* > 0.05).

**Table 1:** Comparison of preoperative parameters between the three groups.

IOL	839MP	MF30	ZXR00	P value
N (eyes)	26	26	26	
Age (years)	50.1 ± 8.1 (40–65)	51.6 ± 6.1 (44–61)	51.9 ± 5.8 (43–62)	0.508
Female/male	8/5	11/2	7/6	
AL (mm)	27.91 ± 1.98	28.10 ± 1.27	27.62 ± 1.99	0.803
ACD (mm)	3.47 ± 0.35	3.42 ± 0.22	3.28 ± 0.29	0.385
FLAT K (mm)	37.45 ± 1.91	38.77 ± 1.84	37.51 ± 2.32	0.213
STEEP K (mm)	38.51 ± 2.37	39.60 ± 1.70	38.38 ± 2.37	0.215
PRE-BCVA	0.61 ± 0.16	0.68 ± 0.17	0.73 ± 0.21	0.072
PREHOA cornea	0.16 ± 0.09	0.16 ± 0.08	0.15 ± 0.04	0.366

ACD: anterior chamber depth; AL: axial length; FLAT K: flat keratometry; STEEP K: steep keratometry; PRE-BCVA: preoperative best-corrected visual acuity; PREHOA cornea: preoperative corneal high-order aberrations. Values presented as means ± standard deviations.

### Visual acuity

**Figure 2:**
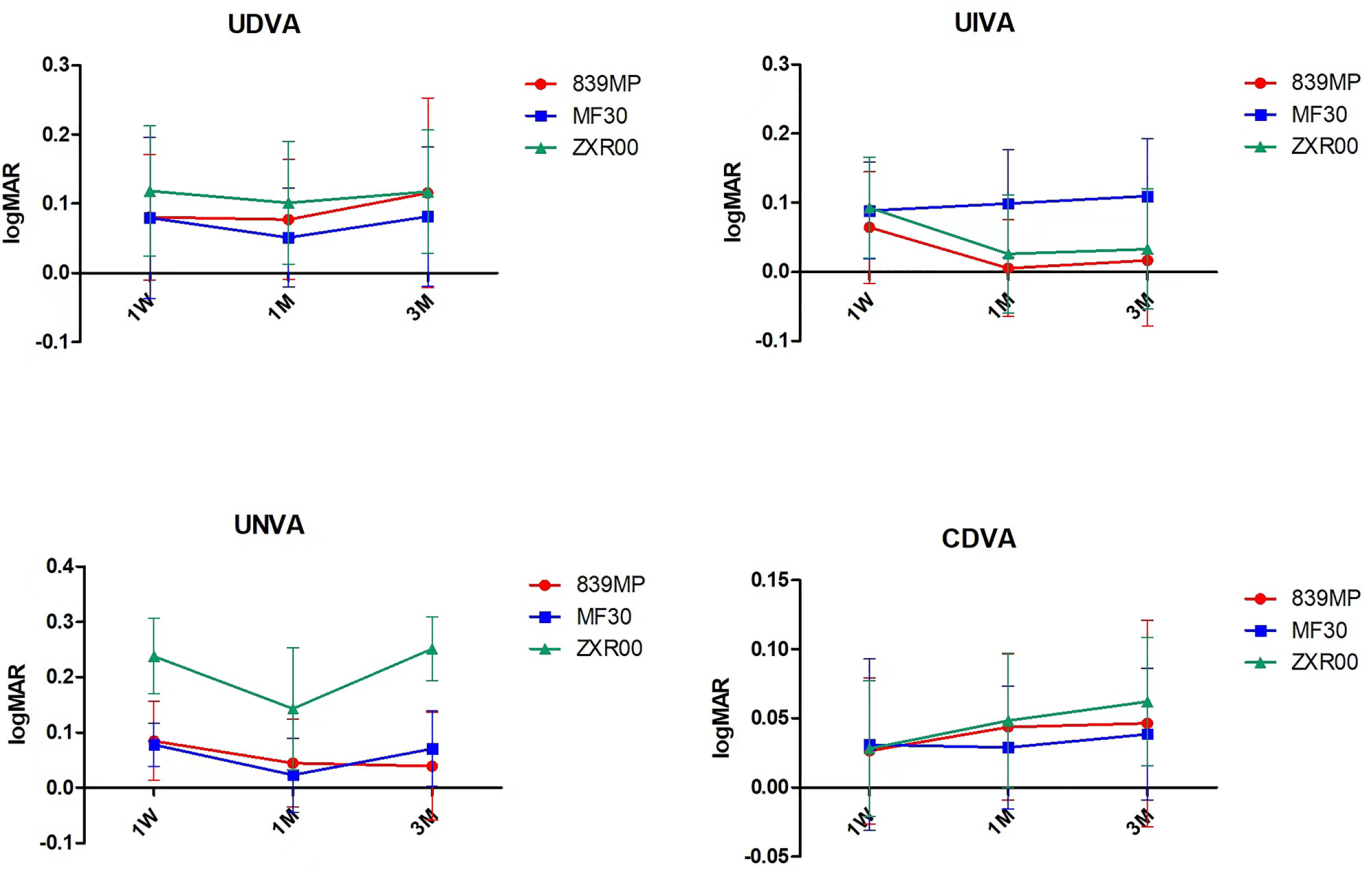
Comparison of the visual acuities of the three groups at different follow-up visits. UDVA: uncorrected distance visual acuity; UIVA: uncorrected intermediate visual acuity; UNVA: uncorrected near visual acuity; CDVA: best-corrected distance visual acuity.

Overall, 87% of the patients in the 3 groups had UDVA more than 0.1 LogMAR. Figure 2 shows the mean UDVA, UIVA, UNVA, and CDVA at each visit. The visual acuities of the three groups were stable at 1 and 3 months after surgery, and there was no difference in UDVA. The difference in UIVA among the three groups was not significant at the 1-week follow-up. However, a significant difference was observed at the 1-month and 3-month follow-up visits, with the UIVA of the MF30 group being significantly lower than that of the 839MP group. The UNVA of the ZXR00 group was the lowest at every follow-up visit. Table 2 shows the intergroup differences in visual acuity. There were significant differences in UIVA between the 839MP and MF30 groups at 1 month (*P* = 0.004) and 3 months (*P* = 0.017) after surgery. The difference in UNVA between the 839MP and ZXR00 groups was statistically significant at 1 week, 1 month, and 3 months after surgery (*P* = 0.000, *P* = 0.007, *P* = 0.000); a similar UNVA difference was found between the ZXR00 and MF30 groups (*P* = 0.000).

**Table 2:** Statistical comparison of visual acuity among the three groups.

P value: 839MP versus MF30			
Parameter	1W Postop	1M Postop	3M Postop
UDVA	0.981	0.441	0.497
UIVA	0.423	0.004*	0.017*
UNVA	0.762	0.450	0.303
CDVA	0.853	0.469	0.712

P value: 839MP versus ZXR00			
Parameter	1W Postop	1M Postop	3M Postop
UDVA	0.271	0.458	0.956
UIVA	0.315	0.455	0.623
UNVA	0.000^+^	0.007^+^	0.000^+^
CDVA	0.939	0.832	0.511

P value: ZXR00 versus MF30			
Parameter	1W Postop	1M Postop	3M Postop
UDVA	0.399	0.169	0.384
UIVA	0.899	0.054	0.052
UNVA	0.000^‡^	0.008^‡^	0.000^‡^
CDVA	0.952	0.349	0.258

CDVA: corrected distance visual acuity; UDVA: uncorrected distance visual acuity; UIVA: uncorrected intermedia visual acuity; UNVA: uncorrected near visual acuity.

*Statistically significant difference between 839MP and MF30.

^+^Statistically significant difference between 839MP and ZXR00.

^‡^Statistically significant difference between ZXR00 and MF30.

### Refractive stability

**Figure 3:**
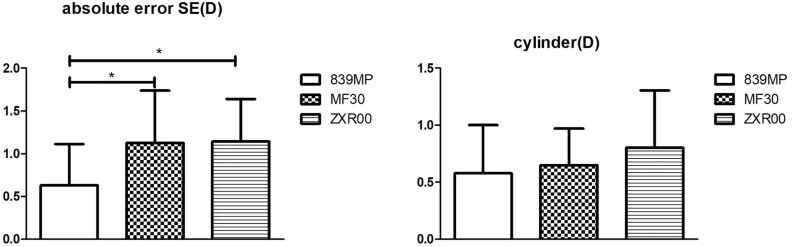
Postoperative refractive stability at 3 months (SE: spherical equivalent).

Figure 3 shows that there was a statistically significant difference in the AESE between the 839MP and MF30 groups (*P* = 0.018) and between the 839MP and ZXR00 groups (*P* = 0.007). However, no significant difference was found between the MF30 and ZXR00 groups. The residual astigmatism did not differ significantly across the three groups (*P* = 0.359).

### High-order aberrations

At a 3.0-mm pupil, the spherical aberrations of the 839MP, MF30, and ZXR00 groups were 0.09 ± 0.12 *μ*, 0.12 ± 0.13 *μ*, and 0.04 ± 0.02* μ*, respectively. At a 4.0-mm pupil, the spherical aberrations of the three groups were 0.33 ± 0.16 *μ*, 0.50 ± 0.08 *μ*, and 0.39 ± 0.10 *μ*, which were significantly higher than that observed in patients who had not undergone LASIK or PRK surgery. However, there were no significant differences in the total eye coma, spherical aberration, and trefoil among the three groups with 3.0-mm and 4.0-mm pupils at 3 months postoperatively (Table 3).

**Table 3:** Comparison of HOA in patients with 3.0-mm and 4.0-mm pupils among the three groups.

Group	3.0-mm pupil			4.0-mm pupil		
	Total HOA coma (μ)	Total HOA sph (μ)	Total HOA trefoil (μ)	Total HOA coma (μ)	Total HOA sph (μ)	Total HOA trefoil (μ)
839MP	0.09 ± 0.04	0.09 ± 0.12	0.04 ± 0.04	0.37 ± 0.21	0.33 ± 0.16	0.35 ± 0.11
MF30	0.08 ± 0.09	0.12 ± 0.13	0.06 ± 0.03	0.47 ± 0.23	0.50 ± 0.08	0.39 ± 0.19
ZXR00	0.06 ± 0.04	0.04 ± 0.02	0.05 ± 0.05	0.42 ± 0.19	0.39 ± 0.10	0.43 ± 0.15
P value	0.204	0.091	0.793	0.131	0.108	0.082

HOA: higher-order aberration.

Values presented as means ± standard deviations.

### Analysis of objective visual quality

There were no significant differences in MTF and SR among the three groups with 3.0-mm and 4.0-mm pupils when compared with the 10 cycles per degree or 30 cycles per degree (*P* > 0.05).

### Visual function questionnaire and spectacle independence

**Figure 4:**
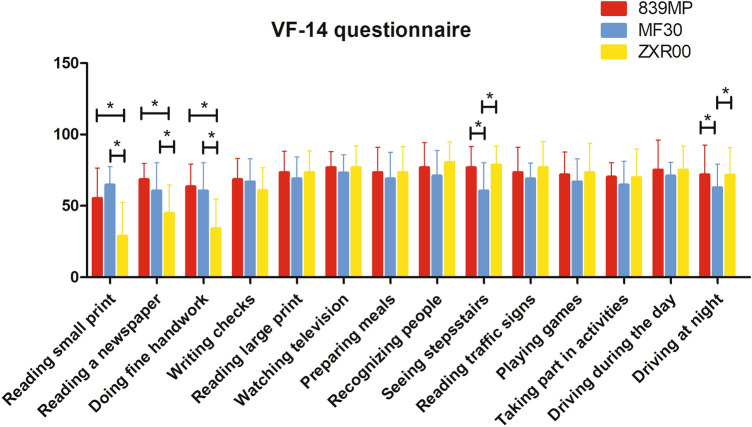
Visual Function Index 14 (VF-14) questionnaire scores for the three groups.

**Figure 5:**
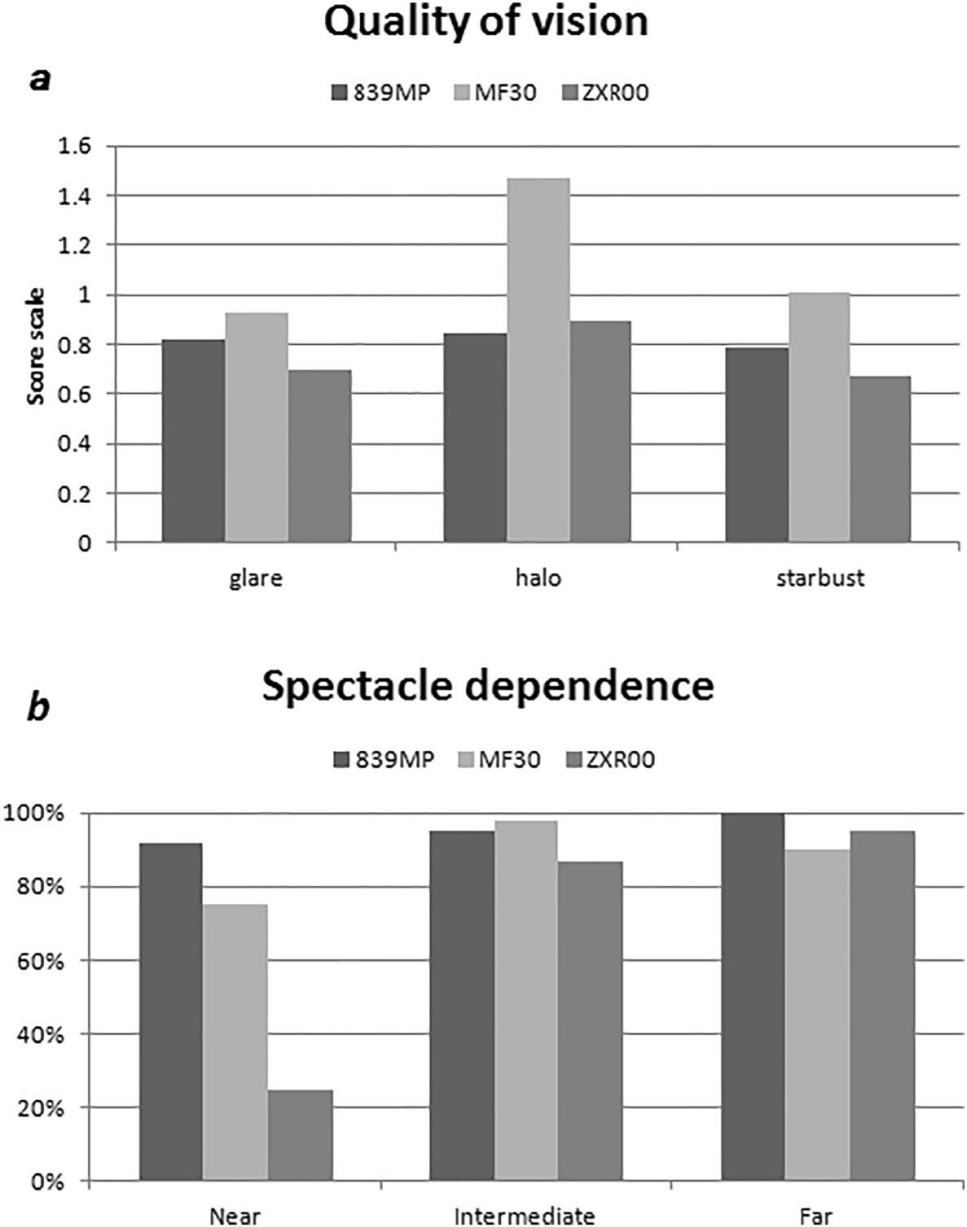
(**a**) Photic phenomena. (**b**) Spectacle dependence in the three groups. IOL: intraocular lens; VA: visual acuity.

Results of the Visual Function questionnaire related to near visual acuity were different among the three groups at the 3-month follow-up (*P* = 0.046). The ZXR00 group scored significantly lower than the other two groups for “Reading small print,” “Reading a newspaper,” and “Doing fine handwork” (*P*_1_ = 0.015, *P*_2_ = 0.000,* P*_3_ = 0.000) based on pairwise comparison. The MF30 group scored lower than the other two groups for “seeing staircase” and “driving at night” (Fig. 4). Figure 5a shows the average score of photic phenomena concerning frequency, severity, and bothersome. There were statistically significant differences in the prevalence of "halos" between the MF30 group and the other two groups (*P*_1_ = 0.039, *P*_2_ = 0.031). Figure 5b shows the spectacle dependence (near, intermediate, and far) in the three groups. The ZXR00 group showed significantly higher near-vision spectacle dependence (*P* < 0.001 vs. 839MP, *P* < 0.001 vs. MF30) at 3 months.

## Discussion

With more accurate formulas for IOL power calculations after LASIK, cataract surgeons have gained confidence in selecting presbyopia-correcting IOLs for patients who have undergone LASIK or PRK. Within the last 5 years, several studies have reported good visual outcomes of presbyopia-correcting IOLs in these patients^8–10^. However, the IOLs were rarely compared. This study further aimed to evaluate the visual quality performance after the implantations of three types of presbyopia-correcting IOLs (839MP, MF30, and ZXR00) after myopic LASIK or PRK for the first time.

The patients in all three groups showed significant improvement in the distance, intermediate, and near visual acuities postoperatively, with UDVA of 0.1 LogMAR or better in 87% of patients. No statistically significant differences in the UDVA and CDVA were found among the three groups at 1 week, 1 month, or 3 months after surgery (P < 0.05). However, the UIVA of patients in the MF30 group was the lowest due to the limitations of design. The ZXR00 group showed the worst near visual acuity, which is related to its lower near addition; this is consistent with the study of Abulafia et al.^14^. Therefore, it is not recommended for patients with high near-vision requirements.

We adopted the Barrett Ture K formula for IOL power calculation with a target refraction of − 0.2 to − 0.75 D; 67% of the patients had a postoperative spherical equivalent error of ± 0.50 D. These results further confirm the accuracy of the Barrett True K formula for IOL calculation in eyes that have undergone laser in-situ keratomileusis surgery, similar to the study by Palomino-Bautista et al.^15^. Moreover, with the lowest AESE, the superior refractive stability of 839MP was validated.

In our study, there were no statistically significant differences in the total eye coma, spherical aberration, or trefoil between the three groups postoperatively. Given that there were no statistically significant differences in age, UDVA, CDVA, AL, corneal curvature, and corneal HOAs before surgery, the difference in visual quality was mainly due to the different optical designs of the IOLs. It is believed that HOA is correlated with some complications such as halo, glare, and suboptimal visual acuity after multifocal IOL implantation^16,17^. However, significant increases in the HOAs, especially spherical aberrations, were observed in patients after keratorefractive surgery. The postoperative changes in spherical aberration in patients after corneal refractive surgery are mainly located in the corneal area of 4–6 mm^18,19^. In our study, the most significant changes in aberration were spherical, with a mean value of 0.63 ± 0.28 *μ* for a 4-mm pupil. This was similar to the observation of Fernández-Vega L et al.^20^. We also found that spherical aberrations within the central 3.0 mm of the cornea did not change significantly. Based on this finding, changes in corneal spherical aberration after LASIK or PRK do not significantly affect the visual quality with presbyopia-correcting IOLs implants in patients without large pupils at night. It is important to pay attention to the range of spherical aberrations in patients after LASIK or PRK. The changes in 3.0–6.0-mm spherical aberrations and the size of the pupil should also be observed. Some studies have reported a significant decrease in overall visual quality with an increase in spherical aberration after myopic LASIK or PRK, and aspheric IOLs can better compensate for the increase in spherical aberration after myopic LASIK or PRK surgery^21^. Similar results were found in our study. The three IOLs (839MP, MF30, and ZXR00) had spherical aberrations of − 0.18 *μ*, 0 *μ*, and − 0.27*μ*, respectively. After surgery, the total eye spherical aberrations of the three groups of patients through a 4-mm pupil were 0.33 ± 0.16 *μ*, 0.50 ± 0.08 *μ*, and 0.39 ± 0.08 *μ*, respectively. The MF30 group was found to have the highest whole-eye spherical aberration postoperatively (0.50 ± 0.08 *μ*). The incidence of photic phenomena (mainly manifesting as halos and glares) was also higher for the MF30 group than for the other two groups, which was shown in the results of our questionnaire. Surgeons should select IOLs with appropriate negative spherical aberrations to compensate for the spherical aberrations induced by LASIK or PRK^4^. Objective visual quality can be quantified using the MTF curve, Strehl ratio (SR), and other indicators^22,23^. The point spread function (PSF) can be directly collected to calculate the SRs using iTrace. The MTF curve, which reflects the different spatial frequencies and clarity of imaging, can also be obtained after the Fourier transform of the PSF. The low-frequency region of the MTF curve reflects the capacity of the optics to transmit the object contour contrast, whereas the high-frequency region shows the detailed transmission capacity resolution. In this study, we used MTF values to evaluate distance visual acuity at a spatial frequency of 10 cycles per degree and near visual acuity at a spatial frequency of 30 cycles per degree. The differences in MTF among the three groups were not statistically significant at 10 cycles per degree and 30 cycles per degree when the pupil diameters were 3.0 mm and 4.0 mm, respectively. This indicated that the three types of IOLs provide comparable daytime visual quality in patients with myopic LASIK or PRK.

The 839MP group had significantly less difficulty for fine visual activities such as reading a paper. This may be attributed to the high near addition of the 839MP IOL. The pupil dependence of MF30 and the low addition design (+ 1.75 D) of ZXR00 accounted for the poor near vision. Among the three groups, patients with 839MP had the highest spectacle independence for both distance and near vision. The patients with ZXR00 implants had a 95% rate of spectacle independence for distance vision but only a 25% rate for near vision, indicating that presbyopic glasses for near vision are still required for most patients. Caution should be exercised in recommending ZXR00 to patients with long axial lengths and good near vision preoperatively. Muñoz et al. reported a 10.6% prevalence of halo in patients with cataracts who had not undergone LASIK or PRK after implantation of MF30 IOL despite the excellent visual outcomes^24^. Lubiński et al. reported that the prevalence of halos in patients who have not undergone corneal refractive surgery after implantation of 839MP was 20% relative to 5% after ZXR00 implantation^25^. In our study, the incidence of halos in the MF30, 839MP, and ZXR00 groups were 59%, 47%, and 39%, respectively. Patients who undergo corneal refractive surgery have a higher incidence of photic phenomena after multifocal IOL implantation than those who do not. Therefore, the MF30 should be recommended with caution for patients who drive at night and work in highly bright environments.

Our study had some limitations. First, the sample size was small, and the results may not be generalizable. Large-scale research is required to validate our results. Second, the postoperative contrast sensitivities were not compared because of the limitations of the examination equipment. Third, the patients were not randomized into three groups because they chose the IOL based on their preferences. Finally, we did not investigate the effect of spherical aberration on corneal refractive surgery because aberrations with large pupils were not considered. Further studies should collect corneal aberration data for different pupil sizes and expand the sample to explore the influence of different pupil sizes on visual quality.

In conclusion, implantations of presbyopia-correcting IOLs are predictable in patients who have undergone LASIK or PRK. The patients showed varying degrees of improvement in distance, intermediate, and near visual acuities and generally high satisfaction postoperatively. Extended depth of focus (EDOF) IOLs are not suitable for patients with high near-vision requirements because of their limited near-vision performance after LASIK or PRK. The change in corneal spherical aberration through a 4-mm pupil after LASIK or PRK leads to an increase in photic phenomena at night. While this does not change MTF under photopic conditions, presbyopia-correcting IOLs, especially MF30, should be implanted with caution in patients with large pupils at night.

## Data Availability

The datasets used and/or analyzed during the current study are available from the corresponding author on reasonable request.
